# Analytical methods matter too: Establishing a framework for estimating maximum metabolic rate for fishes

**DOI:** 10.1002/ece3.7732

**Published:** 2021-07-13

**Authors:** Tanya S. Prinzing, Yangfan Zhang, Nicholas C. Wegner, Nicholas K. Dulvy

**Affiliations:** ^1^ Earth to Ocean Research Group, Department of Biological Sciences Simon Fraser University Burnaby BC Canada; ^2^ Department of Zoology & Faculty of Land and Food Systems University of British Columbia Vancouver BC Canada; ^3^ Fisheries Resources Division Southwest Fisheries Science Center National Marine Fisheries Service (NMFS) National Oceanic and Atmospheric Administration (NOAA) La Jolla California

**Keywords:** active metabolic rate, aerobic metabolism, aquatic respirometry, elasmobranch, maximum exercise, metabolic theory

## Abstract

Advances in experimental design and equipment have simplified the collection of maximum metabolic rate (MMR) data for a more diverse array of water‐breathing animals. However, little attention has been given to the consequences of analytical choices in the estimation of MMR. Using different analytical methods can reduce the comparability of MMR estimates across species and studies and has consequences for the burgeoning number of macroecological meta‐analyses using metabolic rate data. Two key analytical choices that require standardization are the time interval, or regression window width, over which MMR is estimated, and the method used to locate that regression window within the raw oxygen depletion trace. Here, we consider the effect of both choices by estimating MMR for two shark and two salmonid species of different activity levels using multiple regression window widths and three analytical methods: rolling regression, sequential regression, and segmented regression. Shorter regression windows yielded higher metabolic rate estimates, with a risk that the shortest windows (<1‐min) reflect more system noise than MMR signal. Rolling regression was the best candidate model and produced the highest MMR estimates. Sequential regression models consistently produced lower relative estimates than rolling regression models, while the segmented regression model was unable to produce consistent MMR estimates across individuals. The time‐point of the MMR regression window along the oxygen consumption trace varied considerably across individuals but not across models. We show that choice of analytical method, in addition to more widely understood experimental choices, profoundly affect the resultant estimates of MMR. We recommend that researchers (1) employ a rolling regression model with a reliable regression window tailored to their experimental system and (2) explicitly report their analytical methods, including publishing raw data and code.

## Graphical abstract

Advances in experimental design and equipment have simplified the collection of maximum metabolic rate (MMR) data for a more diverse array of water‐breathing animals. However, little attention has been paid to the consequences of statistical choices on the estimation of MMR. We show that MMR estimates are sensitive to the type of statistical analysis used to generate them and provide a framework for model selection.

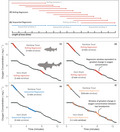

## 1 INTRODUCTION

Metabolic rate is the rate at which organisms convert food and materials from their environment into energy to fuel their biological processes. It is regarded as a fundamental rate of life and a key indicator of physiological performance across tissues, cells and whole organisms (Brown et al., 2004; White & Kearney, 2013). Examination of metabolic rate is becoming increasingly popular within the fields of ecology and comparative physiology as a bridge to link organismal physiology to population, community, and ecosystem phenomena, and to help us understand and make predictions about vulnerable species, diverse ecosystems, and climate change (Barneche et al., 2014; Deutsch et al., 2015; Pörtner et al., 2017). Specifically, recent work has drawn additional attention to the ecological importance of estimating metabolic rate during moderate‐to‐high levels of energy expenditure, including maximum metabolic rate (MMR), which sets the upper ceiling to organismal energy budgets and physiological constraints (Christensen et al., 2020; Deutsch et al., 2015; Killen et al., 2016; Rubalcaba et al., 2020).

MMR is usually defined as the highest aerobic metabolic rate attainable by an organism (Farrell, 2016; Norin & Clark, 2016). In fishes, MMR is typically measured and expressed through the proxy measurement of oxygen consumption following exhaustive exercise or air exposure (Norin & Clark, 2016). The standardization of experimental approaches for estimating MMR is improving as a growing number of studies outline the design and setup of associated respirometry experiments (Cech Jr. & Brauner, 2011; Chabot et al., 2016; Clark et al., 2013; Nelson, 2016; Svendsen et al., 2016). However, the analytical process of actually estimating MMR from the experimental oxygen consumption data immediately following exhaustive exercise or air exposure—specifically, the statistical algorithm used to regress oxygen consumption over time—has not been systematically tested or standardized, despite recent recognition that these analytical choices affect MMR estimates (Little et al., 2020; Norin & Clark, 2016; Zhang et al., 2019, 2020). Often, details concerning the analytical approach used to estimate MMR are not clearly reported, and when provided, there is usually little or no explanation as to why those specific methods were chosen. These unknowns and lack of consistency potentially bias MMR estimates and makes comparison between studies difficult.

Respirometry experiments used to estimate fish MMR typically measure the rate of oxygen depletion from a sealed chamber of water containing the test individual, and then fit a regression to the change in oxygen concentration as a function of time (Svendsen et al., 2016). When estimating MMR, a change in the amount of time over which maximum oxygen consumption is analyzed (specifically, the width of the regression window) may change the slope of this relationship and the resulting MMR estimate (Norin & Clark, 2016). This is because when MMR is measured following a chase to exhaustion protocol, the change in the rate of oxygen consumption over time represents the animal's recovery within the respirometer chamber, which is not perfectly linear as the animal returns to a pre‐exercise oxygen consumption rate over time. Hence, too long a window width can incorporate periods of lower oxygen consumption rate, depressing the MMR estimate. Conversely, noise, brief spikes, and inherent error in experimental systems set a minimum limit on window width because the metabolic rate signal must be large enough to be detectable against the background noise of the experimental system itself, necessitating that the respirometer chamber size be matched to the size of the individual fish (Zhang et al., 2019).

Despite these considerations, there is currently no widely accepted method for selecting a suitable regression window width over which to determine MMR. Window widths vary across studies and may even go unreported; 1–5 min is common, but much longer windows are not unusual (e.g., 10 and 15 min) (Killen et al., 2007; Závorka et al., 2018). In some cases, the window width is tailored to each individual and thus varies across individuals within a study (Slesinger et al., 2019). However, the degree to which MMR estimates are affected by the choice of regression window width, and under what experimental conditions, is unknown.

**FIGURE 1:**
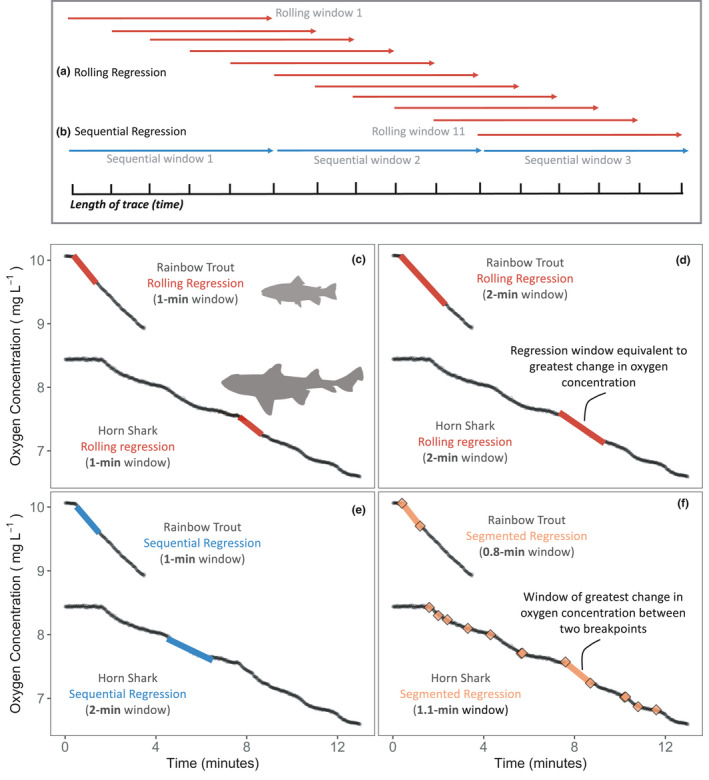
Conceptual schematic of the sampling window for (a) rolling and (b) sequential regression windows and (c‐d) the application of rolling, (e) sequential, and (f) segmented regressions to raw oxygen data used to estimate maximum metabolic rate. (a) Rolling regression windows overlap by one timestep estimating all possible Ordinary Least Squares regressions across the oxygen consumption trace. (b) Sequential regression windows have no overlap and line up end‐to‐end across the oxygen consumption trace. (c‐f) Raw oxygen consumption traces of example individual Rainbow Trout (0.088 kg body mass, 2.25 L chamber volume) and California Horn Shark (1.7 kg body mass, 30.2 L chamber volume) over time showing where the respective model estimates the regression window to occur. (c‐d) Rolling regression with a 1‐ and 2‐min regression window, respectively, (e) sequential regression with a 1‐min window for Rainbow Trout and a 2‐min window for California Horn Shark, and (f) segmented regression with estimated breakpoint locations indicated by colored points. (a) and (b) are inspired by Figure 2 in Harianto et al. (2019)

Two common analytical methods exist for analyzing MMR data in aquatic respirometry: rolling regression and sequential regression. Rolling regression is growing in popularity because the overlapping regression windows give an extremely high resolution that reduces the chance of missing the MMR window, and this method is simple to implement with common programming software such as Excel, R, and Labchart (Figure 1a) (Harianto et al., 2019; Zhang et al., 2019). By comparison, sequential regression is a more conventional method and works by placing regression windows of a set width end‐to‐end along a set of raw oxygen depletion data, limiting the placement of each regression window to a much smaller subset within the oxygen depletion trace (Figure 1b; Tirsgaard et al., 2015; Zhang et al., 2019). In addition to these commonly used methods, a third model, segmented regression (sometimes termed broken‐stick regression), may be useful in taking advantage of the generally unstable nature of oxygen depletion traces immediately postexercise (Figure 1f). This model is typically used to estimate hypoxia tolerance or critical oxygen tension in aquatic ectotherms but has not previously been used to estimate MMR (Reemeyer & Rees, 2019; Slesinger et al., 2019). For this model, we hypothesized that the beginning and end of each MMR window would be marked by a change in the rate of oxygen consumption, detectable as “breakpoints” that define the unique location and width of the MMR window for each individual. However, like window width itself, the suitability and effect of each of these models for the estimation of MMR has yet to be thoroughly tested.

Here, we first estimated MMR and its time‐point within the oxygen depletion trace for two shark and two salmonid species using each of three analytical methods: rolling regression, sequential regression, and segmented regression. This allowed us to compare how applicable each analytical method may be across a variety of life histories: an inactive benthic shark, a demersal shark of medium activity level, and two relatively high activity level pelagic salmonids. We estimated MMR using multiple window widths within both rolling and sequential regression models to test for the effect of window width on MMR estimate. Second, we compared the resulting MMR estimates from all models within and across each species. Third, because the relationship between metabolic rate and body mass is foundational to the theories behind aerobic scope and metabolic ecology (Bigman et al., 2021; Brown et al., 2004; Clark et al., 2013), we also tested the effect of model choice on the allometry of MMR and body mass for the California Horn Shark (*Heterodontus francisci*, our inactive benthic shark species), for which data were collected over a wide body size range. We sought a model that (1) was easily applied to data from a variety of species, (2) relied on the least amount of subjective decision making, and (3) produced MMR estimates with reasonably low variance across individuals. Ultimately, this work demonstrates the importance of considering analytical methods when estimating MMR and provides a framework with which to approach such analyses.

## 2 METHODS

We collated maximum metabolic rate (MMR) data sets from a sedentary benthic elasmobranch, the California Horn Shark, *Heterodontus francisci* Girard 1855 (*n =* 17, 0.203–4.44 kg, data from Prinzing et al., in prep), a demersal shark of medium activity, the Gray Smoothhound, *Mustelus califonicus* Gill 1864 (*n =* 4, 0.76–1.6 kg), and two highly active salmonid species, the Rainbow Trout ﻿*Oncorhynchus mykiss* (Walbaum 1792) (*n =* 16, 0.06–0.11 kg, data from Zhang et al., 2020), and Atlantic Salmon *Salmo salar* Linnaeus 1758 (*n =* 20, 0.06–0.12 kg, data from Zhang et al., 2016). Data for sharks were collected using relatively large individuals across a wide body size range, while data for salmonids were collected using relatively small juveniles. All data were collected using intermittent flow respirometry and a chase‐to‐exhaustion protocol (see Appendix for further detail). Each protocol yielded in a single oxygen depletion trace for each individual, the entirety of which was used for further analysis (e.g., Figure 1c‐f). We then used each of three analytical methods to estimate MMR for each individual: (1) rolling regression with 1‐ to 5‐min sampling window widths, (2) sequential regression with 1‐ and 2‐min window widths, and (3) segmented regression. MMR was estimated by fitting a regression model (see specifics for each model below) to different windows of time across the oxygen consumption trace and searching for the steepest slope. The slope of this regression ($\alpha_{O_{2}}$) was then used to calculate oxygen consumption ($M_{O_{2}}$) using the equation.

(1) $$M_{O_{2}}=\left[\left(V_{r}-V_{f}\right)\times \alpha_{O_{2}}\right]/M_{f}$$

where *V*_r_ is the respirometer chamber volume in liters, and *V*_f_ is the fish volume (assumed to be equivalent to the fish mass, *M*_f_). Additionally, we estimated the time‐point at which MMR occurred along each individual oxygen consumption trace. All statistical analyses were carried out in R (R version 3.6.3 [2020‐02‐29]) and corresponding raw data and code are available online (see Data Availability). The *R* ^2^ values of all regressions used to generate MMR estimates were above 0.9, and in most cases above 0.95.

Using simulated background respiration data, we tested the effectiveness of a signal‐to‐noise ratio analysis method in determining an appropriate regression window width to use in the analysis of MMR data (Zhang et al., 2019, 2020). This method leverages the low ratio of oxygen consumption signal relative to system noise detectable in a background respiration trace to inform the minimum window width that may be appropriate for that experimental system. The method is unique in our field as the only attempt we know of to precisely estimate a minimum regression window width, and though we were unable to confirm the effectiveness of this method, we felt it was important to share these findings to encourage additional testing. Details and results of this analysis are included in the Appendix.

### 2.1 Rolling regression

A rolling regression model runs all possible Ordinary Least Squares regressions of a specified window width across a set of data, stepping forward by one data point at a time (Figure 1a) (Harianto et al., 2019). This removes the chance of missing the period of highest oxygen consumption within the data set. For example, a ten‐min oxygen depletion trace, where oxygen concentration was measured every second, would result in 541 1‐min or 481 2‐min regression estimates.

We applied a rolling regression model across the full measurement cycle for each individual by applying the function roll_regress() from the *rollRegres* package (Christoffersen, 2019, version 0.1.3). This model results in a dataset of regression coefficients, one row for each individual regression. From this, we selected the single regression window producing the steepest slope coefficient and used this to estimate MMR with Equation (1). We used this model to estimate MMR for each of 1‐, 2‐, and 3‐min regression window widths for salmonids (their oxygen consumption was measured over a shorter, 3.5‐ to 4.5‐min time period), and 1‐, 2‐, 3‐, and 5‐min regression window width for sharks (their oxygen consumption was measured over a longer 10–12 min time period), thus producing three estimates of MMR for salmonids and four for sharks. These window widths were chosen as they are commonly used to study MMR in fishes and allowed us to compare the effects of window width on MMR estimation (Auer et al., 2018; Norin & Clark, 2016; Roche et al., 2013).

### 2.2 Sequential regression

MMR was also estimated for each fish using a sequential regression model where regression lines were placed end‐to‐end along each oxygen consumption trace (Figure 1b). For each individual, a 30‐s “lag period” was removed from the beginning of each trace. This lag arises because of the time delay until oxygen‐depleted water expelled from the fish's gills circulates past and is recorded by the oxygen meter probe. The use of a lag period was not necessary for the segmented and rolling regression models because these model's high resolution naturally accounts and adjusts for this lag period. The first regression window was then placed at this corrected start time, using a 1‐min regression window for salmonids and both a 1‐ and 2‐min window for sharks. We were limited by the time over which oxygen consumption was measured for salmonids (3.5–4.5 min) and were only able to use a 1‐min regression window for them in this analysis. Slopes of oxygen consumption over time were estimated for each sequential time window, moving across the MMR trace by one regression window width with no overlapping data used (Figure 1b, e). The regression window yielding the steepest slope was then used to estimate MMR.

### 2.3 Segmented regression

Segmented regression estimates breakpoints that are changes in the relationship between the predictor and response variables, as well as the distance between these points. Applied to respirometry data, a segmented model can estimate breakpoints that represent changes in the rate of oxygen consumption over time, and the distance between each breakpoint gives us a regression window. We ran an iterative segmented regression model on each oxygen depletion trace for each individual to estimate a unique regression window using the segmented() function from the package *segmented* (Muggeo, 2003, 2008, version 1.2.0). The slope of the regression of oxygen consumption as a function of time over this regression window was then used to estimate MMR.

To estimate a regression window for each individual, we repeatedly applied the segmented regression model to each oxygen depletion trace to estimate an iteratively increasing number of breakpoints. The model starts by estimating a single breakpoint in the rate of oxygen consumption over time, then two break points, three, and so on until no more breakpoints can be estimated. Each iteration of the model is a completely independent estimate of the number and locations of breakpoints, meaning they can occur at different locations than in earlier iterations of the model. We used the iteration of the model yielding the sampling window with the steepest slope coefficient to estimate MMR for that individual, irrespective of the total number of breakpoints estimated. Because the segmented regression model estimates breakpoints where it detects a significant change in the rate of oxygen consumption, sometimes placing breakpoints extremely close to one another, it was necessary to specify a minimum acceptable window width to prevent unreasonably high MMR estimates caused by spurious changes in oxygen concentration or measurement error (i.e., background noise). The 90% detection confidence limit reported by the manufacturers of our oxygen meters was 40 s, and this was the only variance within our experimental systems we could quantify confidently. Hence, we set 45 s between breakpoints as a more conservative minimum regression window and we removed slope coefficients from our output data frame that corresponded to window widths shorter than this.

### 2.4 Comparison among models

We tested for the effect of model on MMR estimate within each species. Each MMR estimate was standardized to mean body mass for California Horn Shark (1.95 kg), Rainbow Trout (0.073 kg), and Atlantic Salmon (0.092 kg). To do this, we calculated residual MMR values as the difference between the measured and predicted MMR value within each species according to the relationship between MMR and body mass (MMR *=a M* *^b^*, where *a* and *b* are constants calculated for each model for each species, and *M* is body mass) (Norin et al., 2016; Xiao et al., 2011). Residual values were normally distributed (Shapiro–Wilk, *p* >.05) for all models. For each individual, we then added the raw residual MMR value (positive or negative) to the predicted MMR value at the mean body mass for each species to standardize the absolute MMR to the species‐specific mean body mass. Due to the small number of individuals tested in this study (*n* = 4), Gray Smoothhound were not quantitatively analyzed as we were not able to mass‐standardize their estimates and we instead reported their estimates unstandardized as mass‐specific values.

To test for the effect of model on MMR estimate within each species (California Horn Shark and salmonids), we fit a linear mixed effects model with standardized MMR estimate as a function of model name with individual identity as a random effect (Bates et al., 2015). We then compared between mean values for each model and accounted for multiple comparisons and unequal variance using the emmeans()function (Lenth et al., 2021, version 1.6.0).

Along with each MMR estimate, we estimated the timepoint along the oxygen consumption trace when the MMR window was identified for each individual for each model, measured as time from first placement in the respirometer chamber to the midpoint of each regression window. We tested for the effect of model on window location by fitting a linear mixed effects model with window location as a function of model name with individual identity as a random effect (Bates et al., 2015). We then compared between mean window location values for each model and account for multiple comparisons and unequal variance using the emmeans()function (Lenth et al., 2021, version 1.6.0).

Because California Horn Shark data were collected using animals across a wide body‐size range, we were able to test for the effect of model on the slope estimate of log_10_ MMR as a function of log_10_ body mass. We fit a linear mixed effects model with log_10_ MMR as a function of log_10_ body mass, with model as an interaction term and individual as a random effect, then compared across slope estimates and accounted for multiple comparisons using the emtrends() function (Lenth et al., 2021, version 1.6.0).

## 3 RESULTS

### 3.1 How does the choice of window width and regression model affect the MMR estimate?

**FIGURE 2:**
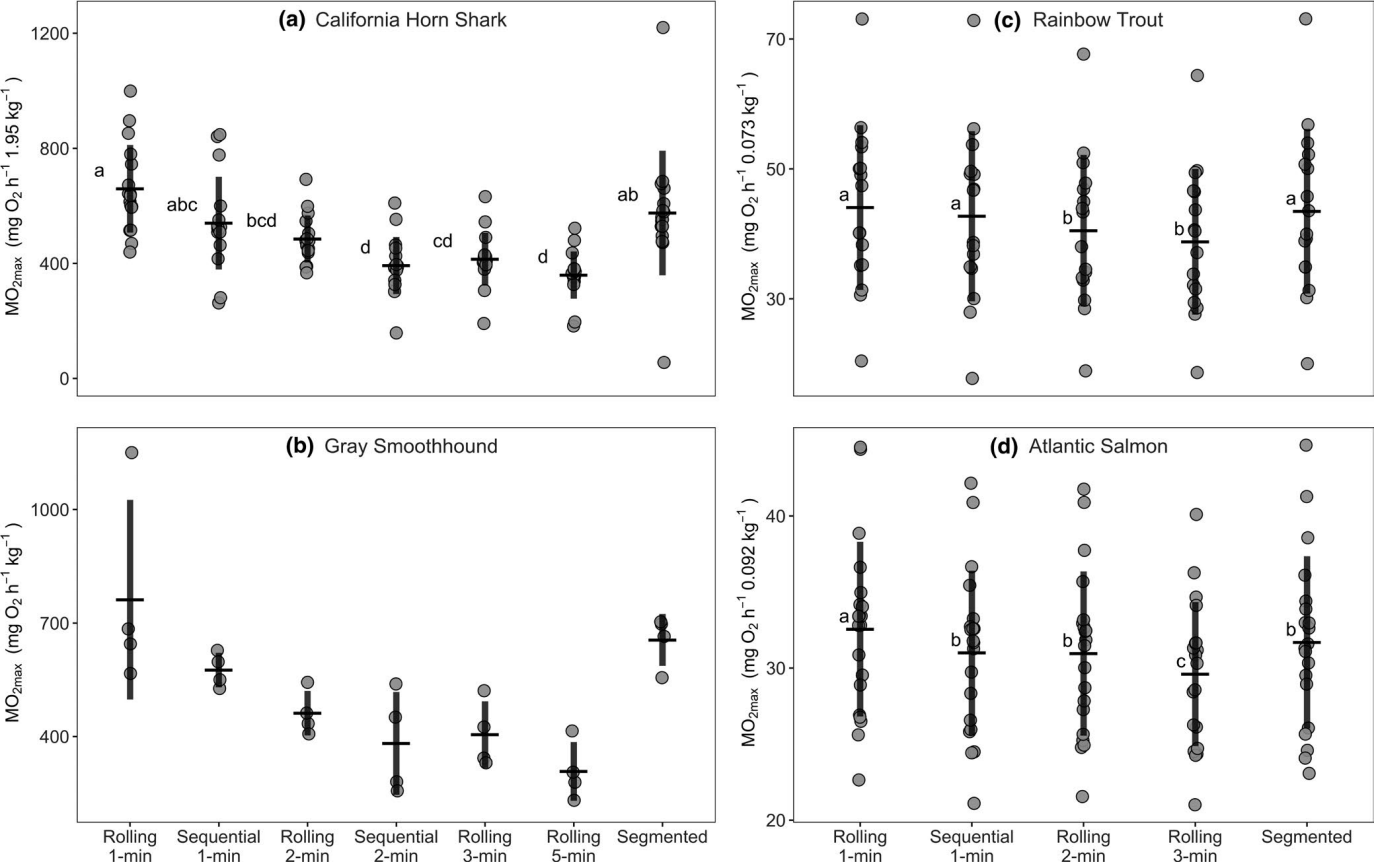
Mean MMR estimate decreased with increasing regression window width. Rolling and sequential regression window width used is indicated by the time listed (e.g., 1‐min) in each model label. Sequential regression model mean MMR estimates were lower than those estimated with equivalent window width rolling regression models in all cases. Unique letters indicate significance level of *p* < 0.05 between compared models. Each species’ MMR estimates were standardized to the mean species mass before analysis (see y‐axis), except Gray Smoothhound which were not mass‐standardized and are reported as mass‐specific values. Means are reported ±SD

**FIGURE A1:**
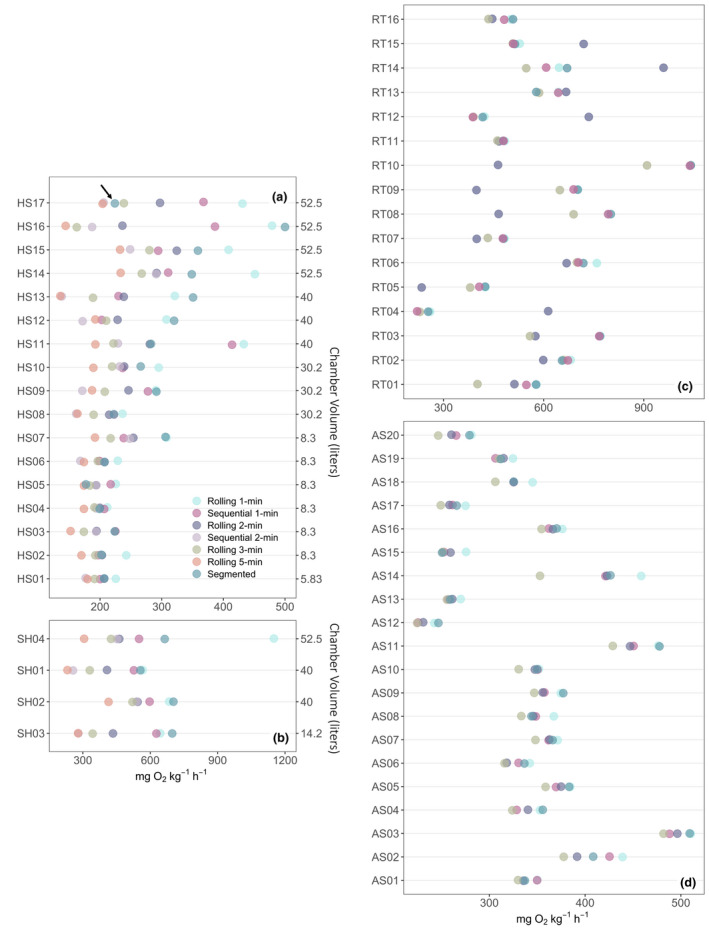
MMR estimates from each model varied within and between individuals in each species: (a) California Horn Shark (HS, 0.203–4.44 kg), (b) Gray Smoothhound (SH, 0.76–1.60 kg), (c) Rainbow Trout (RT, 0.06–0.11 kg), and (d) Atlantic Salmon (AS, 0.06–0.12 kg). The two salmonid species are plotted in order of increasing body mass, beginning with 01. For the two shark species, individuals are plotted in order of increasing body mass within a correspondingly increasing chamber size (see Table A1 for details). Arrow in (a) indicates a segmented regression MMR estimate, which would have doubled if a 41‐s window width, rather than a 45‐s window width, was used. Mass‐specific MMR values are reported for each individual

Shorter regression window widths yielded higher MMR estimates in all species (Figure 2; Figure A1, Table 1). In pairwise comparisons between adjacent models, the largest difference occurred between the shortest window widths, where the 1‐min window rolling regression model mean MMR estimates were 36%, 7%, and 5% higher than the 2‐min window rolling regression model mean MMR estimates for California Horn Shark, Rainbow Trout and Atlantic Salmon respectively (Figure 2). As window width increased, both the relative difference in mean MMR estimate between subsequent models and the standard deviation around the mean MMR estimate decreased.

**TABLE 1:** Shorter regression window widths yielded higher MMR estimates in all species

Model Comparison	California Horn Shark		Rainbow Trout		Atlantic Salmon			Scaling of MMR and body mass for California Horn Shark		
	Estimate	*p*‐value	Estimate	*p*‐value	Estimate	*p*‐value		Estimate	*p*‐value	
Rolling 1‐min ‐ Rolling 2‐min	174.37	0.0010	3.56	0.0001	1.61	0.0000		0.12	0.2043	
Rolling 1‐min ‐ Rolling 3‐min	244.74	0.0000	5.29	0.0000	2.97	0.0000		0.18	0.0093	
Rolling 1‐min ‐ Rolling 5‐min	299.65	0.0000	**–**	**–**	**–**	**–**		0.22	0.0011	
Rolling 1‐min ‐ Segmented	64.95	0.3973	0.52	0.9318	0.74	0.0197		0.05	0.9917	
Rolling 1‐min ‐ Sequential 1‐min	119.19	0.0681	1.34	0.3681	1.57	0.0000		0.06	0.8655	
Rolling 1‐min ‐ Sequential 2‐min	266.65	0.0000	**–**	**–**	**–**	**–**		0.22	0.0010	
Rolling 2‐min ‐ Rolling 3‐min	70.37	0.6135	1.73	0.1411	1.36	0.0001		0.06	0.9014	
Rolling 2‐min ‐ Rolling 5‐min	125.28	0.0465	**–**	**–**	**–**	**–**		0.09	0.5404	
Rolling 2‐min ‐ Segmented	−109.41	0.3087	−3.04	0.0013	−0.88	0.0752		−0.07	0.6134	
Rolling 2‐min ‐ Sequential 1‐min	−55.18	0.8316	−2.22	0.0292	−0.04	0.9998		−0.06	0.9106	
Rolling 2‐min ‐ Sequential 2‐min	92.28	0.2858	**–**	**–**	**–**	**–**		0.09	0.5225	
Rolling 3‐min ‐ Rolling 5‐min	54.91	0.8351	**–**	**–**	**–**	**–**		0.03	0.9952	
Rolling 3‐min ‐ Segmented	−179.79	0.0033	−4.77	0.0000	−2.23	0.0000		−0.13	0.0707	
Rolling 3‐min ‐ Sequential 1‐min	−125.55	0.0456	−3.95	0.0000	−1.40	0.0000		−0.12	0.2435	
Rolling 3‐min ‐ Sequential 2‐min	21.91	0.9983	**–**	**–**	**–**	**–**		0.03	0.9940	
Rolling 5‐min ‐ Segmented	−234.70	0.0000	**–**	**–**	**–**	**–**		−0.16	0.0115	
Rolling 5‐min ‐ Sequential 1‐min	−180.40	0.0006	**–**	**–**	**–**	**–**		−0.15	0.0570	
Rolling 5‐min ‐ Sequential 2‐min	−33.00	0.9843	**–**	**–**	**–**	**–**		0.001	1.0000	
Segmented ‐ Sequential 1‐min	54.24	0.9783	0.82	0.8385	0.83	0.1094		0.01	0.9978	
Segmented ‐ Sequential 2‐min	201.70	0.0005	**–**	**–**	**–**	**–**		0.17	0.0106	
Sequential 1‐min ‐ Sequential 2‐min	147.46	0.0095	**–**	**–**	**–**	**–**		0.15	0.0531	

Note

Estimate indicates relative difference (mg O_2_ hr^‐1^) in mean MMR estimates between compared models with associated *p‐*values indicating significance level of comparison (Tukey post hoc test to correct for multiple comparisons) (left‐most columns). Individual MMR estimates were each standardized to the mean mass of each species before analysis (1.95 kg, 0.073 kg, and 0.092 kg for California Horn Shark, Rainbow Trout and Atlantic Salmon, respectively). Relative difference in log‐log regression slope estimates for each model for California Horn Shark MMR and body mass are shown in the right‐most columns (and see Figure 4). Gray Smoothhounds were omitted from this analysis due to low sample size.

Across all models, the 1‐min window rolling regression model produced the highest MMR estimates in all species, followed by estimates made with the segmented regression model (Figure 2, Figure A1, Table 1). Mean MMR estimates for the segmented regression model were higher than California Horn Shark 2‐min window and Atlantic Salmon 1‐min window sequential regression models; however, there was also considerably higher variance around the California Horn Shark segmented regression mean MMR estimate (Figure 2, Table 1). In all species, the sequential regression models produced lower mean MMR estimates compared to their corresponding rolling regression model with the same window width, where California Horn Shark 1‐ and 2‐min, Rainbow Trout 1‐min, and Atlantic Salmon 1‐min sequential regression model estimates were 22%, 24%, 3%, and 4% lower, respectively (Figure 2, Table 1).

We found larger differences among window widths and models for California Horn Shark than for salmonids (Table 1, Figure 2; Figure A1). California Horn Shark mean body mass was 23 times greater than the mean body mass of the salmonids (1.95 kg and 0.083 kg, respectively), and considerably larger chamber sizes were used to measure oxygen consumption (Table A1). California Horn Shark oxygen consumption traces at larger body masses and chamber sizes were often more variable compared to traces at smaller body masses and to salmonid traces (Figure 1c‐f).

### 3.2 How does timepoint of the MMR window vary?

**FIGURE 3:**
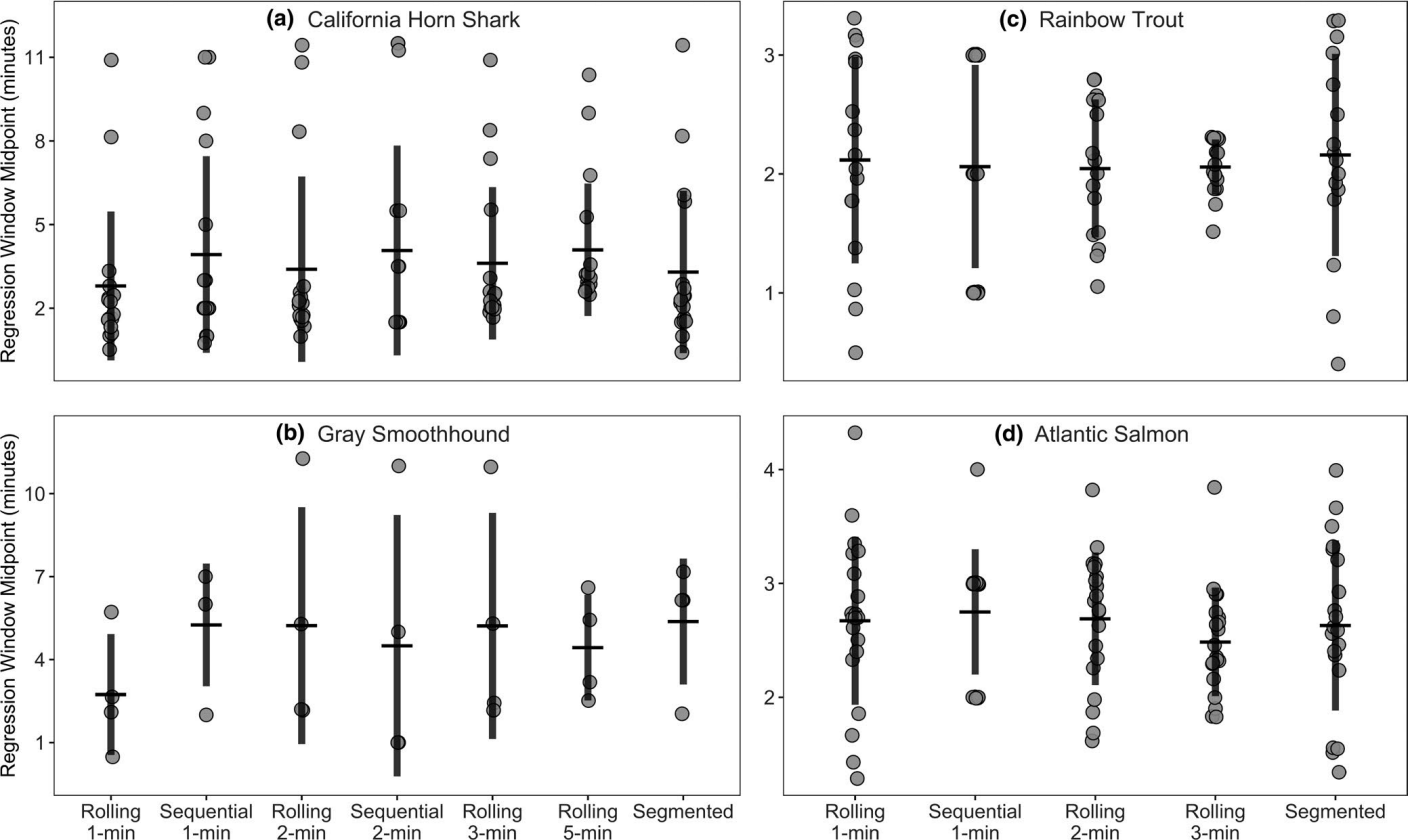
The timepoint of the MMR regression window within the oxygen depletion trace varied between individuals within species but not significantly across models. The timepoint is estimated as the midpoint of the MMR regression window for each model for each individual, measured from when the individual was placed in the respirometer to the midpoint of the MMR regression window. Mean window midpoint is plotted for each model for each species, ±SD

MMR occurred more than two minutes after an individual was placed in the respirometer chamber in 77%, 86%, 63%, and 85% of the California Horn Shark, Gray Smoothhound, Rainbow Trout, and Atlantic Salmon individuals, respectively, with the latest window occurring in a California Horn Shark after 11.5 min (Figure 3). However, 64% of shark MMR windows occurred within the first five min. There was no consistent pattern of variation in window timepoint, and no significant differences between window timepoint mean across models (*p* > 0.14 in all cases).

### 3.3 Does choice of model and window width affect the scaling of MMR and body mass?

**FIGURE 4:**
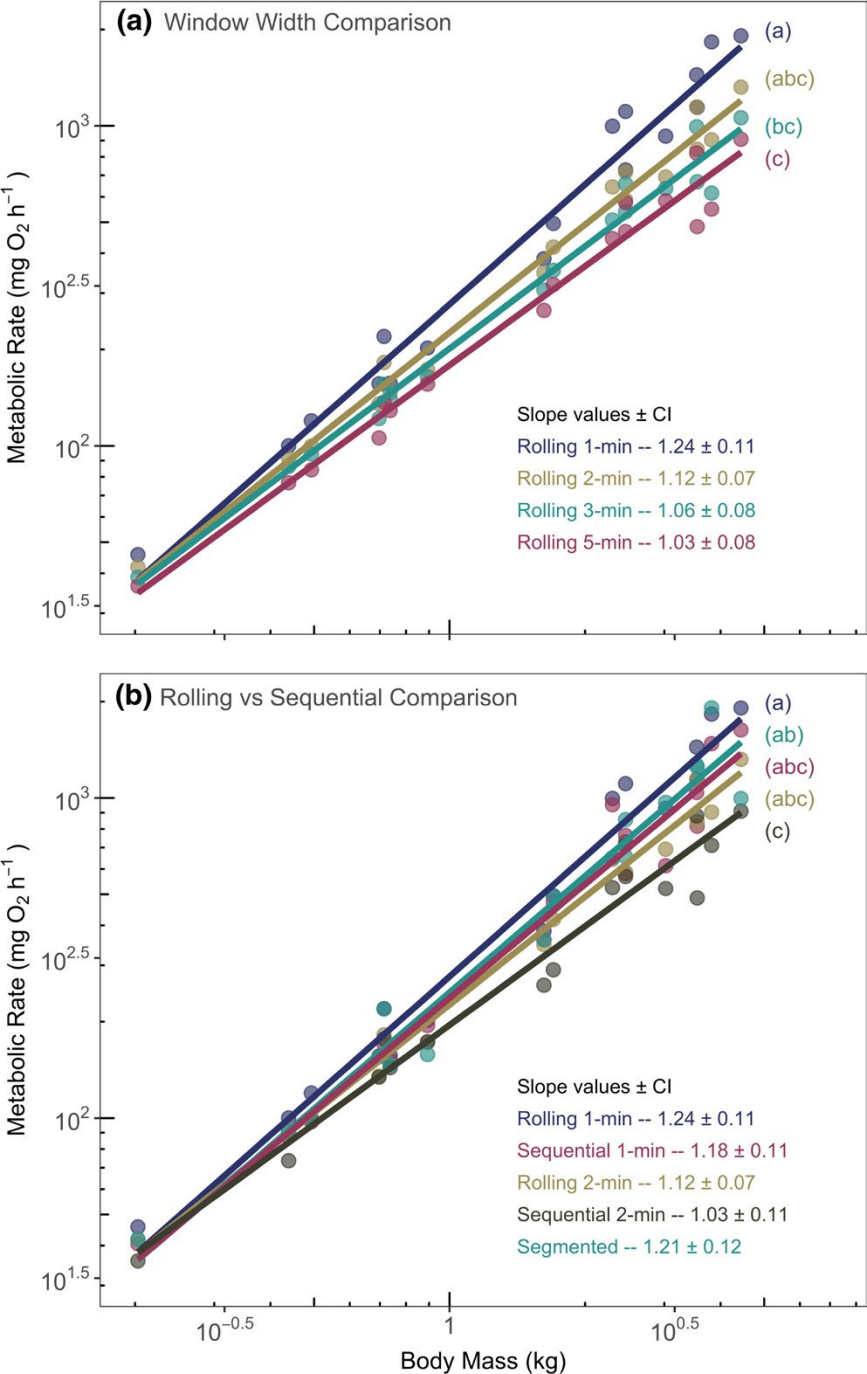
Estimates of absolute MMR plotted as a function of body mass on a log‐log scale for the California Horn Shark for (a) each of the four rolling regression models, and (b) rolling regression models with their corresponding sequential regression models of the same window width and the segmented model. Scaling slope estimates decreased as window width used to generate estimates increased, and rolling regression slope estimates were higher than their corresponding sequential regression slope estimates. Slope estimates are reported ±95% confidence intervals. Letters indicate a significant difference between slope estimates for compared models using a significance level of *p* < 0.05 (and see Table 1)

The choice of regression model had a significant effect on the scaling relationship between MMR and body mass (Table 1, Figure 4). One‐min window rolling regression estimates produced the steepest slope and a relatively wide confidence interval (1.24 ± 0.11 95% CI). Longer window widths resulted in lower estimated slope values; however, this did not significantly reduce confidence intervals (Figure 4a). The 2‐min window rolling regression model MMR estimates resulted in the regression slope estimate with the narrowest confidence interval (1.12 ± 0.07 95% CI) (Figure 4a).

## 4 DISCUSSION

Across four species of varying activity level and body mass, we found that (1) smaller regression windows yielded higher estimates of MMR, (2) MMR was best estimated using a rolling regression model with a 1‐ to 2‐min window, and (3) the time‐point at which MMR occurs is often at least two minutes into the oxygen depletion trace and, hence, may be missed with certain analytical methods, such as with sequential regression or too short of a postexercise monitoring period. This study highlights the necessity of including thorough and detailed analytical methods in the design of respirometry experiments and cautions against directly comparing estimates made with extremely disparate experimental and analytical methods. Here, we outline the key considerations in applying these findings in the analysis of fish respirometry data.

### 4.1 Choosing a window width

In all cases, MMR estimates were sensitive to the window width used in analysis. All regression models required at least a minimum window width be chosen in order to estimate MMR and this choice remains somewhat subjective. If too short a window is used, MMR may be overestimated due to spurious non‐oxygen consumption variance in the system. However, attempting to guard against this with too long of a window width may unnecessarily underestimate MMR without adding significant variance‐handling benefits. At a minimum, raw traces of oxygen depletion over time should be visually checked to get a sense for how reasonable each potential window width and corresponding MMR estimate may be. Spurious changes in oxygen consumption, such as the example California Horn Shark trace in Figure 1c‐f, may be the result of the individual shifting within the respirometer chamber, affecting the mixing of water and potentially altering the curvature of the slope. Traces such as these may require longer window widths compared to more linear traces to account for these obvious nonlinear sections, but with a potential trade‐off of an underestimated MMR. As an additional test, when California Horn Shark MMR estimates were regressed as a function of body mass, the 2‐min window rolling regression estimates produced the smallest confidence interval around the MMR to body‐mass scaling slope estimate of all our tested models (slope = 1.12 ± 0.07; Figure 4). This suggests that this slightly longer window width may be more appropriate for this data set to account for and reduce the influence of higher system variance at larger body masses and respirometry chamber sizes. However, we cannot exclude the possibility that the true estimates of MMR are highly variable across individuals. At this time, we recommend using the same window width across all individuals in a study for consistency.

While we found a negative relationship between MMR estimate and widow width in all species, this effect was considerably weaker in the small salmonids than the much larger sharks (compare Figure 2a,b vs. c,d). This suggests that estimates made using different, but similar, window widths may be more comparable across studies in which relatively small body masses and chambers were used, while studies utilizing different window widths for larger animals and larger chambers may be less comparable. During analysis, multiple window widths should be compared before deciding on the best width for the experimental system, as we have done here. Methods to estimate system‐specific regression window widths show promise, however, our test of the signal‐to‐noise ratio method showed that this method was unable to differentiate between simulated experimental systems to produce a reliable regression window width (see Appendix) (Zhang et al., 2019). Future studies should test whether chamber or fish size is related to MMR estimate by examining if the slope of oxygen concentration over time (i.e., the rate of oxygen depletion itself, before correcting for system volume) is related to fish size or the chamber‐to‐fish volume ratio. Unfortunately, our small sample size at each body mass for each species prevented us from exploring this further.

When comparing between models and window lengths, it is important to note that we chose not to use R^2^ as a tool to assess model fit as it is not a reliable metric for making comparisons between models, especially when the differences in R^2^ values are so small. When data points are added to a model, such as in the case of a 2‐min rather than a 1‐min window width, the R^2^ value will almost always be higher for the model with more data points, even if the model is a worse fit in reality (McElreath, 2015). Additionally, R^2^ is regarded as useful only for assessing general fit of a model (and in combination with other metrics), rather than as a tool for comparing between models. Specifically, it cannot be used to compare the fit of two models that each use unique data sets, such as two oxygen consumption over time regression models where one spans across a window at 2–3 min and the other spans across 4–5 min within an oxygen depletion trace.

### 4.2 Choosing an analytical method

We recommend rolling regression be used to estimate MMR in aquatic systems. The rolling regression model proved to be the most versatile and precise method for estimating MMR and worked well across all species and experimental systems. Its overlapping intervals mean this model has the resolution to test every possible regression within the oxygen depletion data set, greatly reducing the chance of missing the MMR window and making it unnecessary to select a lag period to remove from the beginning of the trace. Statistical software packages such as *respR* make it simple to implement this model on raw data output from a wide variety of oxygen sensing equipment and improve reproducibility across studies (Harianto et al., 2019).

In contrast, the sequential regression model performed poorly. By placing the regression windows end‐to‐end, the low resolution of these models consistently underestimated MMR compared to rolling regression models using the same window width (Figures 1b, 2; Table A1). Specifically, sequential regression may miss the true MMR window if it occurs partially across two successive regression widows. For example, for a 3‐min long MMR trace, a sequential regression model can only produce three 1‐min regression estimates while rolling regression would produce 121 estimates, providing a view of oxygen consumption rate at every single timepoint during the oxygen depletion trace. It has been argued that sequential regression may be a less subjective analysis method because its low resolution may decrease the chance of selecting a window which represents more measurement error than true oxygen consumption. However, measurement error may still fall within the limited window options of sequential regression, possibly resulting in more variability across estimates. We believe that this is a significant drawback to using sequential regression, and that the risk of over‐estimating MMR with rolling regression should be controlled for in other ways. Specifically, we suggest the researcher use rolling regression to compare window widths and select a window width which produces high estimates of MMR while maintaining low variance around the mean estimate between multiple respirometry runs on the same or different individuals.

Finally, the segmented regression model was unable to consistently produce reasonable MMR estimates across individuals, as seen through the large variation in estimates across individuals in comparison with other models (Figure 2; Figure A1). Selecting a minimum allowable regression window width for segmented regression was highly subjective, and in one case, allowing a 41‐s rather than a 45‐s window would have doubled the resulting MMR estimate (Figure A1). Spurious changes in oxygen consumption rate, especially in the larger respirometer chambers, led to the estimation of breakpoints at timepoints where there likely was not a true significant change in the rate of oxygen consumption (Figure 1e).

### 4.3 Choosing a monitoring period

Respirometry experiments are often designed to use short, 3‐ to 5‐min monitoring periods (specifically, periods during which oxygen depletion is measured between chamber flush cycles) under the assumption that individuals will be maximumly aerobic during and immediately following strenuous exercise (Brett, 1964; Norin & Clark, 2016; Rummer et al., 2016). Longer monitoring periods may also not be feasible for species with high metabolic rates that rapidly deplete available oxygen within the respirometer (Svendsen, Bushnell, & Steffensen, 2016). While MMR occurred immediately in most individuals, there were many instances where it occurred after a considerable delay and would have been missed if a shorter monitoring period was used (Figure 3). An extreme case of delayed MMR was found by Clark et al. (2012) in Coho Salmon (*Oncorhynchus kisutch*), where MMR peaked up to five hours after exhaustive exercise. To have the best chance of catching the MMR window, an effort should be made to use the longest monitoring period possible for the tested species and experimental system. For example, measurement of oxygen depletion for California Horn Shark was ended once oxygen concentration within the respirometer chamber reached 80%, after which the flush pump was turned on, allowing for a long initial monitoring period while also protecting the individual against hypoxia. However, a long monitoring period should not be sought at the expense of significantly increasing the chamber‐to‐fish volume ratio, as this will reduce the strength of the oxygen depletion signal and increase the likelihood of measurement error.

### 4.4 Macroecological implications

When plotted against body mass on a log‐log scale, the MMR estimates made with each model for California Horn Shark revealed a pattern of decreasing slope coefficients with increasing regression window widths (Figure 4). This pattern suggests that MMR estimates may not be comparable across studies where significantly different analytical methods were used to generate them, such as 1‐ versus 5‐min regression window widths, especially in larger individuals. However, more work is needed to investigate the consequences of grouping estimates made with potentially disparate analytical methods. Glazier (2005) highlighted that standard metabolic rate estimates for the same species can vary between studies but that it is unclear how much of this is the result of variation across individuals or variation in study design. Because each of our models was tested on the same raw data, we have strong support that the analytical method itself is likely a strong contributor to variation observed between studies using different analytical techniques. We suggest MMR analysis method be considered, in addition to the standard practice of accounting for experimental protocol and temperature, when collating data in future meta‐analyses (Killen et al., 2016).

### 4.5 Conclusions

Despite the rise in appreciation for metabolic ecology and the experimental methods required to estimate metabolic rate, the choice of analytical method has remained largely unstandardized. The implications of the choice of analytical methods are far‐reaching, from the quality of empirical studies and theoretical models, to the comparability of results across species and metabolic ecology's potential as a predictive tool (Deutsch et al., 2015; Glazier, 2009). Additionally, precise estimates of MMR are crucial to understanding species’ response to thermal extremes through the lens of aerobic scope, defined as an animal's capacity for activity above rest (Farrell, 2016). We strongly encourage the use of systematic testing of MMR window‐width as outlined in this paper and the use of rolling regression models in future MMR studies. Finally, we recommend authors report their analytical choices by following principles of reproducible code and data archiving so that future meta‐analyses can more accurately assess interspecific relationships and produce reliable results (Croucher et al., 2017).

## CONFLICT OF INTEREST

The authors declare no conflict of interest.

## AUTHOR CONTRIBUTION

**Tanya Prinzing:** Conceptualization (lead); Data curation (lead); Formal analysis (lead); Methodology (lead); Visualization (lead); Writing‐original draft (lead); Writing‐review & editing (lead). **Yangfan Zhang:** Conceptualization (equal); Data curation (equal); Methodology (supporting); Writing‐review & editing (supporting). **Nicholas C. Wegner:** Data curation (supporting); Funding acquisition (equal); Project administration (supporting); Resources (equal); Supervision (supporting); Writing‐original draft (supporting); Writing‐review & editing (supporting). **Nicholas K. Dulvy:** Conceptualization (supporting); Formal analysis (supporting); Funding acquisition (lead); Methodology (supporting); Project administration (equal); Resources (equal); Supervision (lead); Writing‐original draft (supporting); Writing‐review & editing (supporting).

## Figures and Tables

**FIGURE A2:**
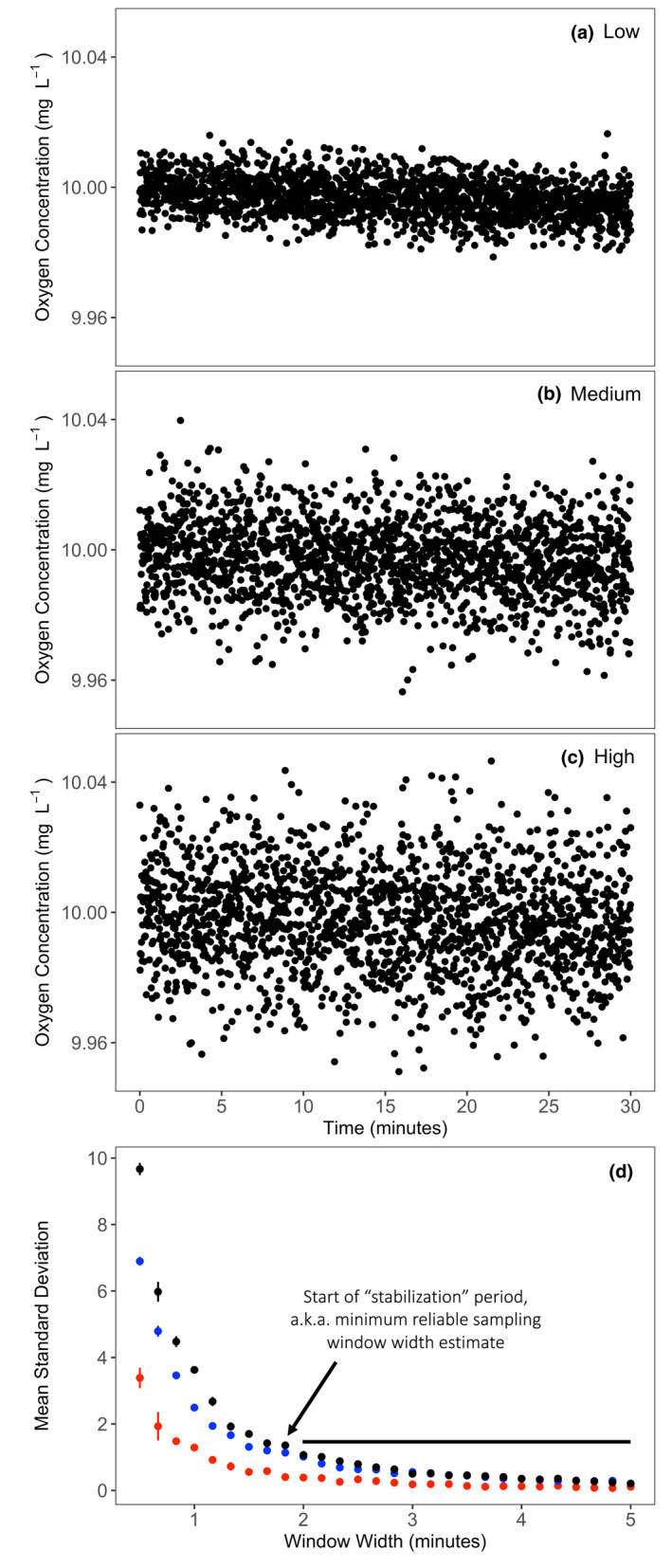
(a–c) Examples of low (mean SD = 0.0055), medium (mean SD = 0.0115), and high (mean SD = 0.0155) noise background respiration traces simulated for signal‐to‐noise ratio analysis. (d) Using a sample size of 8 simulated background respiration traces at each noise level, the mean standard deviation (±*SE*) at each window width is taken within each noise level and then plotted with high, medium, and low noise color coded as black, blue and red, respectively. Horizontal line indicates stabilized section for high‐noise model only, and the window width one above the first stabilized window width is designated as the minimum reliable sampling window width, indicated by the arrow

**FIGURE A3:**
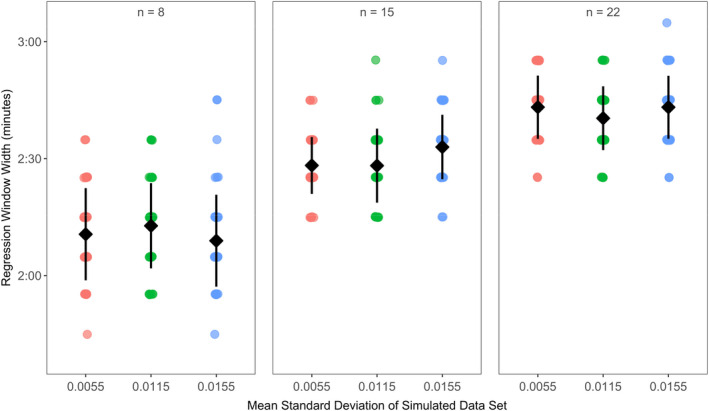
Minimum reliable regression window estimates for each background respiration trace sample size at each noise level. Sample sizes indicate the amount of background respiration traces used to generate each minimum reliable regression window estimate (each data point). The mean minimum reliable regression window width of the 50 total estimates made for each sample size and noise level (±SD) is indicated in black

## Data Availability

All data and code are archived on Dryad at DOI https://doi.org/10.5061/dryad.j0zpc86dn

## APPENDIX 1

### Animal acquisition

Elasmobranchs were caught between June and October 2019 as bycatch during yearly gillnet surveys near San Diego, California, and by hand using SCUBA (Prinzing et al., in prep). Upon capture, individuals were transported to the Southwest Fisheries Science Center (SWFSC) Experimental Aquarium in aerated coolers with frequent water changes to maintain oxygen saturation and reduce waste buildup. Individuals were allowed to acclimate to captivity until they resumed regular feeding and for at least two weeks before experimentation. Sharks were held in 300 × 150 × 90 cm oval tanks (~3,200 L) continuously fed with fresh filtered and UV sterilized seawater (18 ± 0.5°C, ≥100% oxygen saturation, 33.5‰, salinity). This temperature was chosen as it falls at the middle of the species’ natural range and was within about 1°C of the ocean temperature at which individuals were collected. Individuals were fed to satiation every 3–5 days using human‐grade market squid (*Doryteuthis opalescens*) and mackerel (*Scomber japonicus*) and were fasted for a minimum of 48 hr before experiments to remove the influence of specific dynamic action on metabolic rate estimates. Experimental conditions for Rainbow Trout and Atlantic Salmon can be found in Zhang et al. (2020, 2016), respectively.

### Collection of oxygen consumption data

All experiments were carried out using a chase‐to‐exhaustion protocol where each individual was manually chased by hand in a tank large enough to allow unimpeded burst‐swimming (Norin & Clark, 2016; Zhang et al., 2020, 2016). The focal individual was deemed exhausted once it stopped bursting away and began resting on the bottom of the chase tank between stimuli (usually after 4–7 min of chasing). Once exhausted, the focal individual was immediately transferred from the chase tank to the respirometer chamber (Loligo systems, Tjele, Denmark). This process was practiced and optimized so that transfer from the chase tank to respirometer chamber and the start of oxygen consumption measurement took less than 20–30 s. Oxygen consumption rate was measured for a minimum of 10 min for California Horn Shark and Gray Smoothhound, ending once the dissolved oxygen concentration reached 80%. For Rainbow Trout and Atlantic Salmon, a standard, short measurement period protocol was followed, and oxygen consumption was measured over a shorter 3.5‐ to 4.5‐min period. This corresponds to the closed or measurement period, after which the flush valve was opened to flush the chamber with new, fully oxygenated water during the flush period. Background respiration was measured in empty respirometer chambers immediately after an $M_{O_{2}\max}$ trial and was found to be negligible in all cases.

Water was mixed inside the respirometer chamber using a recirculating closed‐loop system with a water pump (Eheim, Deizisau, Germany), and a fiber‐optic oxygen probe was fixed in the recirculation loop to measure dissolved oxygen once every second (Svendsen, Bushnell, & Steffensen, 2016). Horn Shark and Smoothhound respirometry data were collected using Fibox 3 and Fibox 4 oxygen meters and probes (PSt3 Oxygen Dipping Probe, PreSens Precision Sensing GmbH, Germany), and Rainbow Trout and Atlantic Salmon data were collected using FireSting oxygen meters (FSO2‐4 optical oxygen and temp meter FireStingO2) and fiber optic probes (Robust Oxygen Probe OXROB10, PyroScience GmbH, Aachen, Germany). All dissolved oxygen measurements were converted to units of mg/L using the *respR* oxygen unit conversion function (Harianto et al., 2019), accounting for temperature and atmospheric pressure. All experiments were carried out on fasted, laboratory acclimated fish. Because fish species varied in size, multiple respirometer chambers were used to appropriately match the chamber volume to each fish’s body mass. Teleost experiments were carried out using 2.25–2.26 L respirometer chambers only. For California Horn Shark experiments, the range of chamber sizes was 5.825 L–52.5 L with a mean chamber‐to‐fish volume ratio of 15.27. For Gray Smoothhound, the range of chamber sizes was 14.2 L–52.5 L with a mean chamber‐to‐fish volume ratio of 38 (Table A1).

### Signal‐to‐noise ratio method to estimate a regression window width

We tested the effectiveness of a signal‐to‐noise ratio analysis method to estimate a minimum reliable sampling window (regression window) required for the estimation of MMR (Zhang et al., 2019). We did this by simulating background respiration data to represent a hypothetical ideal system. Specifically, within an empty respirometer chamber, the oxygen consumption signal is usually very low and stable over time compared to the system noise. We used iteratively increasing regression window widths to compare system noise to this relatively low oxygen consumption signal of background respiration within the respirometer chamber. From this, we hoped to estimate a minimum reliable sampling window (regression window), which could then be applied to estimate MMR using oxygen consumption data (Figure A2).

To estimate a minimum reliable sampling window, the model begins by running a series of sequential regression models across an individual background respiration trace. Each model iteration uses increasingly larger regression windows, beginning with a short window and increasing incrementally to a set large window (we used 30 s to 5 min). For a 30‐min trace, this results in 60 30‐s regression windows, decreasing to six 5‐min regression windows by the last iteration of the model. Oxygen consumption rate is estimated for each regression window for each model iteration on the background respiration trace. All estimates for each regression window width are then pooled within that window width to estimate a mean oxygen depletion rate with corresponding standard deviation (SD) and coefficient of variation (CV) of that mean. This process is repeated using each individual background respiration trace in the set of background respiration traces being analyzed (detailed below). Then, all SD and CV estimates from all background respiration traces are pooled to estimate a mean SD and mean CV for each window width. Mean SD and mean CV are then each regressed as a function of window width to estimate a minimum reliable sampling window (Figure A2d).

To test this method, we simulated 8, 15, and 22 30‐min background respiration data sets within a hypothetical low, medium, and high noise experimental system, resulting in 9 individual combinations of sample size (8, 15, or 22) and noise level (low, medium, or high) (Figure A2a–c). Each background respiration trace was simulated by sampling one dissolved oxygen value per second from a normal distribution with a resulting average standard error of 0.0055, 0.0115, and 0.0155 for low, medium, and high noise systems, respectively. These sample sizes were chosen as they are similar to those often used in aquatic respirometry experiments, and we used the noise levels within the real background respiration data sets for our tested species to guide us in simulating our hypothetical background respiration data sets. In addition to testing whether the model could differentiate between noise levels, simulating data allowed us to test for the effect of background respiration trace sample size (8, 15, and 22) on the resulting minimum reliable sampling window width estimate. Fifty iterations of each of the 9 individual sample size‐noise level combinations were run resulting in a total of 450 individual minimum reliable sampling window estimates. Because the real rate of background respiration in respirometer chambers varies slightly between trials, slopes of hypothetical oxygen concentration over time were sampled from a uniform distribution of −0.0002 to −0.0022 to allow slight variance between each simulated data frame. The minimum reliable sampling window width was defined as the shortest window width for which mean SD was not statistically significantly different from the mean SD at the longest tested window width (5 min), also known as where the window width stabilizes (Figure A2d). We focused on just SD as an indicator because CV estimates should only be used for data on a ratio scale or data that do not exhibit negative values. Our mean CV estimates were negative in some cases, due to the low signal‐to‐noise ratio of our simulated data, and thus unusable.

### Results of simulation tests

Estimates of the minimum reliable regression window were sensitive to the sample size of the background respiration traces (8, 15, 22), where larger sample sizes resulted in longer window width estimates (ANOVA, *p* < .05) (Figure A3). There was significant variability in window width estimates across iterations of the model; however, this was slightly reduced with larger sample sizes. Mean minimum window width estimates were not significantly different across variance levels, at any sample size. We thus conclude that the model was unable to detect a difference in variance between experimental systems (ANOVA, *p* > .05), and thus could not accurately estimate a minimum window width for any of our simulated experimental systems.

Because we were not able to effectively estimate a minimum reliable regression widow using our simulated data, we were not able to apply this method to our real background respiration traces and report a minimum reliable regression window width for each species/system. However, this method is unique in our field as the only attempt (so far) to precisely estimate a regression window width and we felt it was important to share our negative results for the simulated data analysis. Thus, we reported our findings here in the Appendix of the manuscript.

**TABLE A1:** Body mass, respirometer chamber volume, and chamber‐to‐fish volume ratio for each individual California Horn Shark (HS) and Gray Smoothhound (SH)

Fish ID	Body mass (kg)	Chamber volume (L)	Chamber to fish volume ratio
HS01	0.203	5.8	28.7
HS02	0.439	8.3	18.9
HS03	0.493	8.3	16.8
HS04	0.697	8.3	11.9
HS05	0.715	8.3	11.6
HS06	0.738	8.3	11.2
HS07	0.893	8.3	9.3
HS08	1.62	30.2	18.6
HS09	1.70	30.2	17.8
HS10	2.46	30.2	12.3
HS11	2.30	40.0	17.4
HS12	3.02	40.0	13.2
HS13	3.55	40.0	11.3
HS14	2.46	52.5	21.3
HS15	3.54	52.5	14.8
HS16	3.82	52.5	13.7
HS17	4.44	52.5	11.8
SH03	0.660	14.2	21.5
SH02	0.72	40.0	55.6
SH01	0.907	40.0	44.1
SH04	1.62	52.5	32.4
